# Nonlinear features of the superconductor–ferromagnet–superconductor φ_0_ Josephson junction in the ferromagnetic resonance region

**DOI:** 10.3762/bjnano.13.97

**Published:** 2022-10-21

**Authors:** Aliasghar Janalizadeh, Ilhom R Rahmonov, Sara A Abdelmoneim, Yury M Shukrinov, Mohammad R Kolahchi

**Affiliations:** 1 Department of Physics, Institute for Advanced Studies in Basic Sciences (IASBS), P.O. Box 45137-66731, Zanjan, Iran; 2 BLTP, JINR, Dubna, Moscow Region, 141980, Russia; 3 Dubna State University, Dubna, 141980, Russia; 4 Moscow Institute of Physics and Technology, Dolgoprudny, 141700, Moscow Region, Russia; 5 Physics Department, Menofiya University, Faculty of Science, 32511, Shebin Elkom, Egypt

**Keywords:** Duffing oscillator, Josephson junction, Landau–Lifshitz–Gilbert equation

## Abstract

We demonstrate the manifestations of nonlinear features in magnetic dynamics and *I*–*V* characteristics of a φ_0_ Josephson junction in the ferromagnetic resonance region. We show that at small values of the system parameters damping, spin–orbit interaction, and Josephson-to-magnetic energy ratio, the magnetic dynamics is reduced to the dynamics of a scalar Duffing oscillator driven by the Josephson oscillations. The role of the increasing superconducting current in the resonance region is clarified. Shifting of the ferromagnetic resonant frequency and the reversal of its damping dependence due to nonlinearity are demonstrated by the full Landau–Lifshitz–Gilbert–Josephson system of equations and in its different approximations. Finally, we demonstrate the negative differential resistance in the *I*–*V* characteristics and its correlation with the fold-over effect.

## Introduction

The coupling of the superconducting phase difference with the magnetic moment of a ferromagnet in a φ_0_ junction leads to a number of unique features important for superconducting spintronics and modern information technology [1–5]. It allows one to control the magnetization precession by the superconducting current and affects the current–voltage (*I*–*V*) characteristics by magnetic dynamics in the ferromagnet, in particular, to create a DC component in the superconducting current [6–8]. A remarkable manifestation of this coupling is the possibility to stimulate a magnetization reversal in the ferromagnetic layer by applying a current pulse through the φ_0_ junction [3,9–13].

There are two features of Josephson junctions that come into play in our study. The first one is the broken inversion symmetry in the weak link of the Josephson junction when the link is magnetic, which introduces an extra phase in the current–phase relation, preventing it from being antisymmetric. Such Josephson junctions are named φ_0_ junctions [1], and examples, such as MnSi and FeGe, exist. The second one is the nonlinear property of the system, which makes for an anomalous resonance behavior [14].

We couple such a Josephson junction to the model that describes the magnetodynamics in thin films or heterostructures to form the Landau–Lifshitz–Gilbert–Josephson model (LLGJ) [14–16]. It is shown that, for a particular set of parameters, the coupled equations reduce to the dynamics of a Duffing oscillator [14]. The cubic nonlinearity in this oscillator describes several effects in other models, too [17]. One example are the resonance effects in the antiferromagnetic bimeron in response to an alternating current, which has applications in the detection of weak signals [15,18–19].

The Gilbert damping term is added phenomenologically to the Landau–Lifshitz model to reproduce the damping of the precessing magnetic moment. Gilbert damping is also important in modeling other resonance features, as its temperature dependence affects them [20–21], and, in turn, in the superconducting correlations that affect it [22]. The magnetization precession in an ultrathin Co_20_Fe_60_B_20_ layer stimulated by microwave voltage under a large angle requires modeling by a Duffing oscillator, too. This is aided by the so-called fold-over features, again due to nonlinearity [16,23–24].

The consequences of the nonlinear nature of the coupled set of the LLGJ system of equations in the weak coupling regime was demonstrated recently in [14]. We showed that, in this regime where the Josephson energy is small compared to the magnetic energy, the φ_0_ Josephson junction is equivalently described by a scalar nonlinear Duffing equation. An anomalous dependence of the ferromagnetic resonant frequency (FMR) on the increase of the Gilbert damping was found. We showed that the damped precession of the magnetic moment is dynamically driven by the Josephson supercurrent and the resonance behavior is given by the Duffing spring. The obtained results were based on numerical simulations. The role of the DC superconducting current and the state with negative differential resistance (NDR) in the *I*–*V* characteristics were not clarified. Also, the effects of the Josephson-to-magnetic energy ratio and the spin–orbit coupling (SOC) were not investigated at that time.

In the present paper, we study the nonlinear aspects of the magnetic dynamics and *I*–*V* characteristics of the φ_0_ Josephson junction in the ferromagnetic resonance region. We compare description of the anomalous damping dependence (ADD) exhibited by full LLGJ system of equations with approximated equations and demonstrate the Duffing oscillator features in the small parameter regime. Effects of the Josephson-to-magnetic energy ratio, and the spin–orbit coupling on the ADD, referred to earlier as the α-effect [14] are demonstrated. By deriving the formula that couples the DC superconducting current and maximal amplitude of magnetization we discuss the correlation of superconducting current and the negative differential resistance in the resonance region. Finally, we discuss the experimentally important features by emphasizing the details of the magnetization dynamics and the *I*–*V* characteristics of the φ_0_ junction.

We have shown that, in the limit of small values for the system parameters Josephson-to-magnetic energy ratio *G*, damping α, and spin–orbit coupling *r*, the dynamics is given by a Duffing spring [14]. We focus on the shift in resonance and the effects of nonlinear interactions. We give semi-analytic models to explain our results in various limits.

The paper is organized as follows. In section “Models and Method” we outline the theoretical model and discuss the methods of calculations. The ferromagnetic resonance and the effect of the system parameters on the anomalous damping dependence are considered in subsection A of section “Results and Discussion”. In subsection B we present an analytical description of the dynamics and *I*–*V* characteristics of the φ_0_ junction at small system parameters. The manifestation of negative differential resistance in the *I*–*V* characteristics through the fold-over effect is discussed. We compare the description of the anomalous damping dependence by the full LLGJ system of equations with approximated equations and show how the Duffing oscillator captures the nonlinearities in the regime of parameters with small values in subsection C. We present results on the critical damping and derive a formula that couples the DC superconducting current and the maximal amplitude of magnetization in the ferromagnetic layer. The section “Conclusion” concludes the paper.

## Models and Method

**Figure 1:**
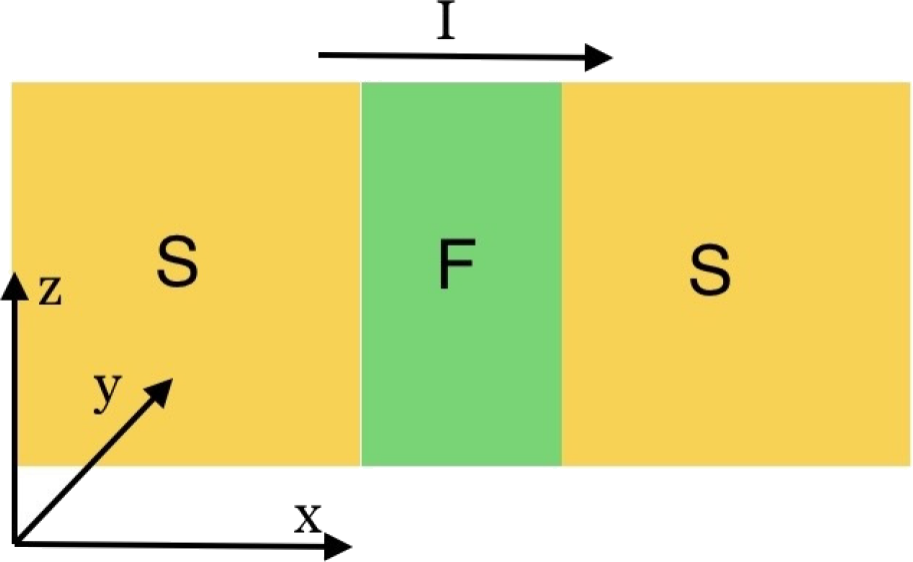
Schematic view of a SFS φ_0_ Josephson junction. The external current is applied along the *x* direction. The ferromagnetic easy axis is along *z* direction.

The following section is closely related to our work in [13]. The φ_0_ junction [6,12,25] that we study is shown in Figure 1. The current–phase relation in the φ_0_ junction has the form *I*_s_ = *I*_c_sin(φ − φ_0_), where φ_0_ = *rM_y_*/*M*_0_, *M_y_* denotes the component of magnetic moment in the 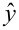 direction and *M*_0_ is the modulus of the magnetization. The physics of φ_0_ Josephson juncton is determined by a system of equations, which consists of the Landau–Lifshitz–Gilbert (LLG) model, the resistively capacitively shunted junction (RCSJ) model expression with the current–phase relation (*I*_s_) described above, and the Josephson relation between phase difference and voltage.

The dynamics of the magnetic moment **M** is described by the LLG equation [26]:


[1]

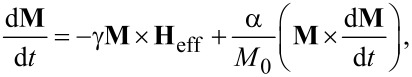



where **M** is the magnetization vector, γ is the gyromagnetic relation, **H**_eff_ is the effective magnetic field, α is the Gilbert damping parameter, and *M*_0_ = |**M**|.

In order to find the expression for the effective magnetic field we have used the model developed in [6], where it is assumed that the gradient of the spin–orbit potential is along the easy axis of magnetization taken to be along 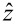. In this case the total energy of the system can be written as


[2]

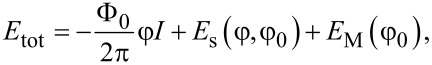



where φ is the phase difference between the superconductors across the junction, *I* is the external current, *E*_s_(φ,φ_0_) = *E*_J_[1 − cos(φ − φ_0_)], and *E*_J_ = Φ_0_*I*_c_/2π is the Josephson energy. Here Φ_0_ is the flux quantum, *I*_c_ is the critical current, *r* = *l*υ_so_/υ_F_, *l* = 4*hL*/ℏυ_F_, *L* is the length of the ferromagnetic (F) layer, *h* is the exchange field of the F layer, 
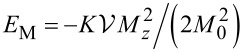
, the parameter υ_so_/υ_F_ characterizes a relative strength of spin–orbit interaction, *K* is the anisotropic constant, and 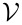 is the volume of the F layer.

The effective field for LLG equation is determined by


[3]

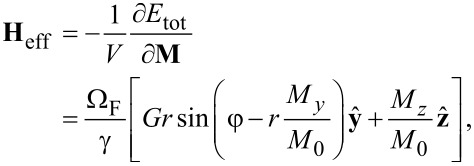



where Ω_F_ = γ*K*/*M*_0_ is the frequency of the ferromagnetic resonance and 
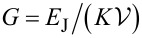
 determines the ratio between Josephson energy and magnetic energy.

In order to describe the full dynamics of the φ_0_ junction the LLG equations should be supplemented by the equation for the phase difference φ, that is, the equations of the RCSJ model for bias current and the Josephson relation for voltage. According to the extended RCSJ model, which takes into account derivative of φ_0_ phase shift, the current flowing through the system in underdamped case is determined by


[4]

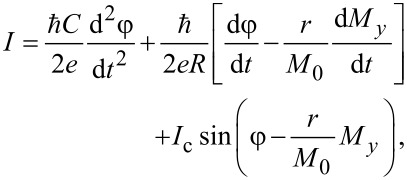



where *I* is the bias current and *C* and *R* are capacitance and resistance of the Josephson junction, respectively. The Josephson relation for the voltage is given by


[5]

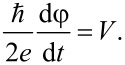



We note that, in the framework of the RCSJ model, the displacement current is proportional to the first derivative of the voltage (or the second derivative of the phase difference). The magnetization dynamics plays the role of an external force, and the first order derivative of φ_0_ is a source of an external current in the JJ. This was demonstrated in [25,27], where the authors included the first derivative of φ_0_ as the source of the electromotive force. The voltage is determined by the phase difference and does not depend on φ_0_. From this point of view, in the framework of the RCSJ model, the external current source cannot modify the expression for the displacement current. This is why we do not include the second derivative of φ_0_ in our model.

Using Equation 1, Equation 3, Equation 4, and Equation 5 we can write a system of equations, in normalised variables, that describes the dynamics of the φ_0_ junction:


[6]

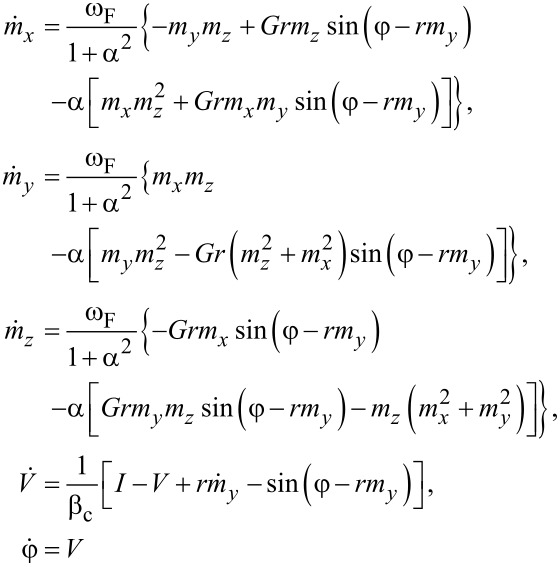



where *m_x,y,z_* = *M_x,y,z_*/*M*_0_ and satisfy the constraint 
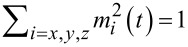
 and β_c_ = 2*eI*_c_*CR*^2^/ℏ is the McCumber parameter. In order to use the same time scale in the LLG and RCSJ equations, in this system of equations we have normalized time to 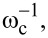 where 
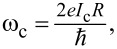
 and ω_F_ = Ω_F_/ω_c_ is the normalized frequency of ferromagnetic resonance Ω_F_ = γ*K*/*M*_0_. The bias current is normalized to the critical current *I*_c_ and the voltage *V* is normalized to *V*_c_ = *I*_c_*R*. The system in Equation 6 is solved numerically using the fourth-order Runge–Kutta method [14].

## Results and Discussion

### A. Effect of system parameters on the anomalous damping dependence

ADD of the FMR frequency with increasing α was discussed in [14]. It was found that the resonance curves demonstrate features of a Duffing oscillator, reflecting the nonlinear nature of the LLGJ system of equations. There is a critical damping value at which anomalous dependence comes into play. This critical value depends on the system parameters. Here, we present the details of such a transformation from usual to anomalous dependence with variations in the spin–orbit coupling and the Josephson-to-magnetic energy ratio.

**Figure 2:**
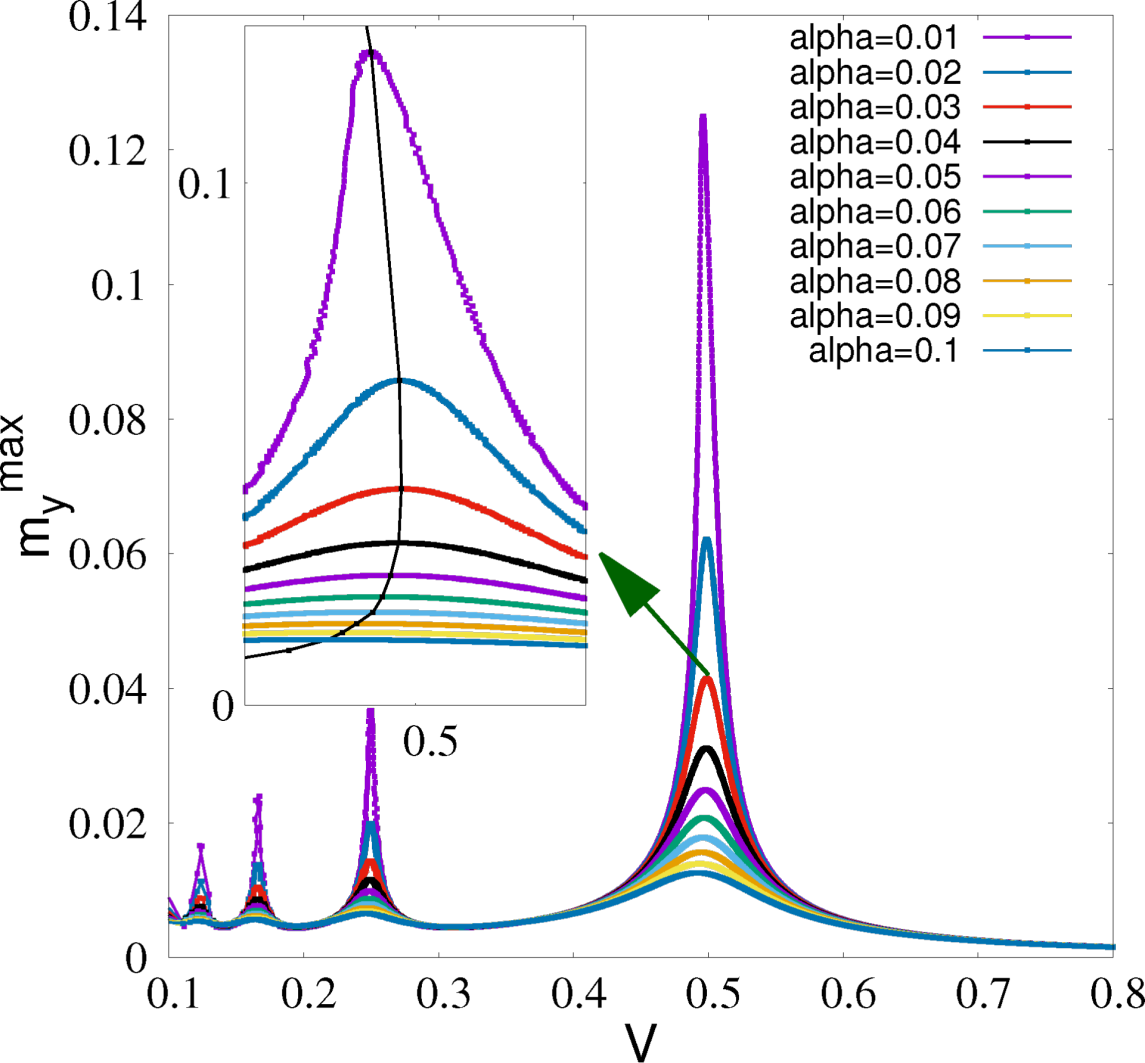
Maximal amplitude of magnetization *m_y_*-component at each value of voltage along the *I*–*V* characteristics of the φ_0_ junction in the ferromagnetic resonance region for different α. The inset enlarges the main maximum. Parameters: β_c_ = 25, *G* = 0.05, *r* = 0.05, and ω_F_ = 0.5.

To investigate the effect of damping, we calculate the maximal amplitude of the magnetization component *m_y_* taken at each value of the bias current based on the LLGJ system of equations (Equation 6). In Figure 2 we show the voltage dependence of the maximal amplitude 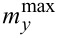 in the ferromagnetic resonance region at different damping parameters and small values of Josephson-to-magnetic energy ratio, *G* = 0.05, and spin–orbit coupling, *r* = 0.05. The ferromagnetic resonance curves exhibit the different forms. An increase in damping shows a nonuniform change in the resonant frequency: It approaches ω_F_ instead of moving away with increase in α. We emphasize that this happens at small *G* and *r*. We consider that such behavior can be explained by the nonlinear nature of the LLGJ system of equations. There is a manifestation of subharmonics of the FMR in Figure 2 at *V* = 0.25, 0.167, and 0.125.

We usually expect the resonance peak to move away from resonance as α increases. Figure 2 shows that this normal effect is accompanied with an anomalous behavior, as can be seen in the inset in this figure, where the resonance peak approaches ω_F_ as α increases [14].

**Figure 3:**
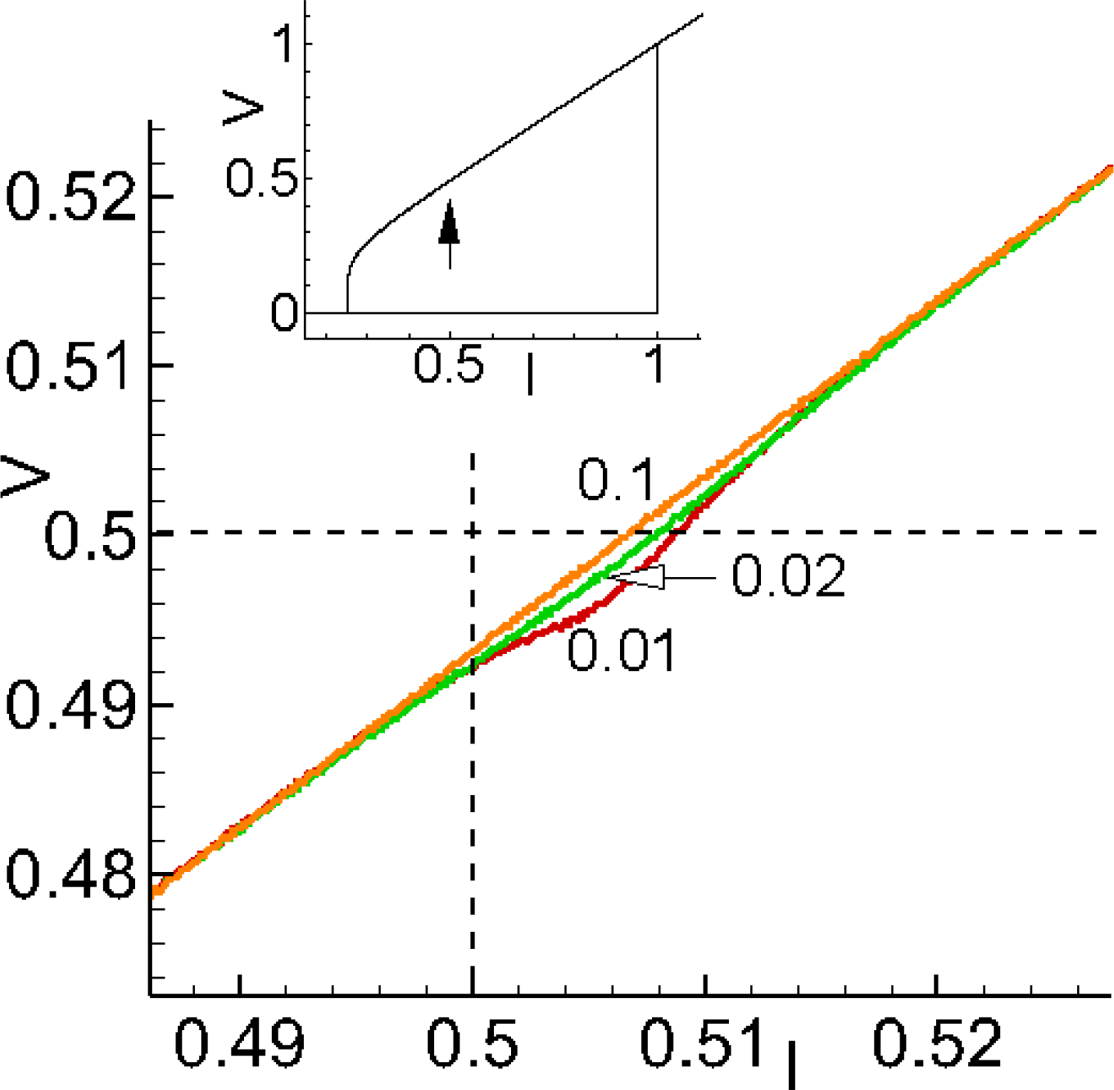
Part of the *I*–*V* characteristics of the φ_0_ junction at *G* = 0.05, *r* = 0.05, and different values of Gilbert damping. The numbers show the α value. The inset shows the total *I*–*V* characteristics and the arrow indicates the resonance region.

The manifestation of FMR in the *I*–*V* characteristics of the φ_0_ junction at three values of the damping parameter is demonstrated in Figure 3. A strong deviation of the *I*–*V* curve is observing at α = 0.01, which is a characteristic value for many magnetic materials. This fact indicates that ADD can be observed experimentally by measuring the *I*–*V* characteristics in wide interval of the damping parameter.

Interesting features of ADD appear through a variation of spin–orbit coupling. As it was demonstrated in [28], an increase in SOC leads to an essential change in *I*–*V* characteristics and magnetization precession in the ferromagnetic resonance region. The nonlinearity goes stronger and a state with negative differential resistance appears at large SOC.

**Figure 4:**
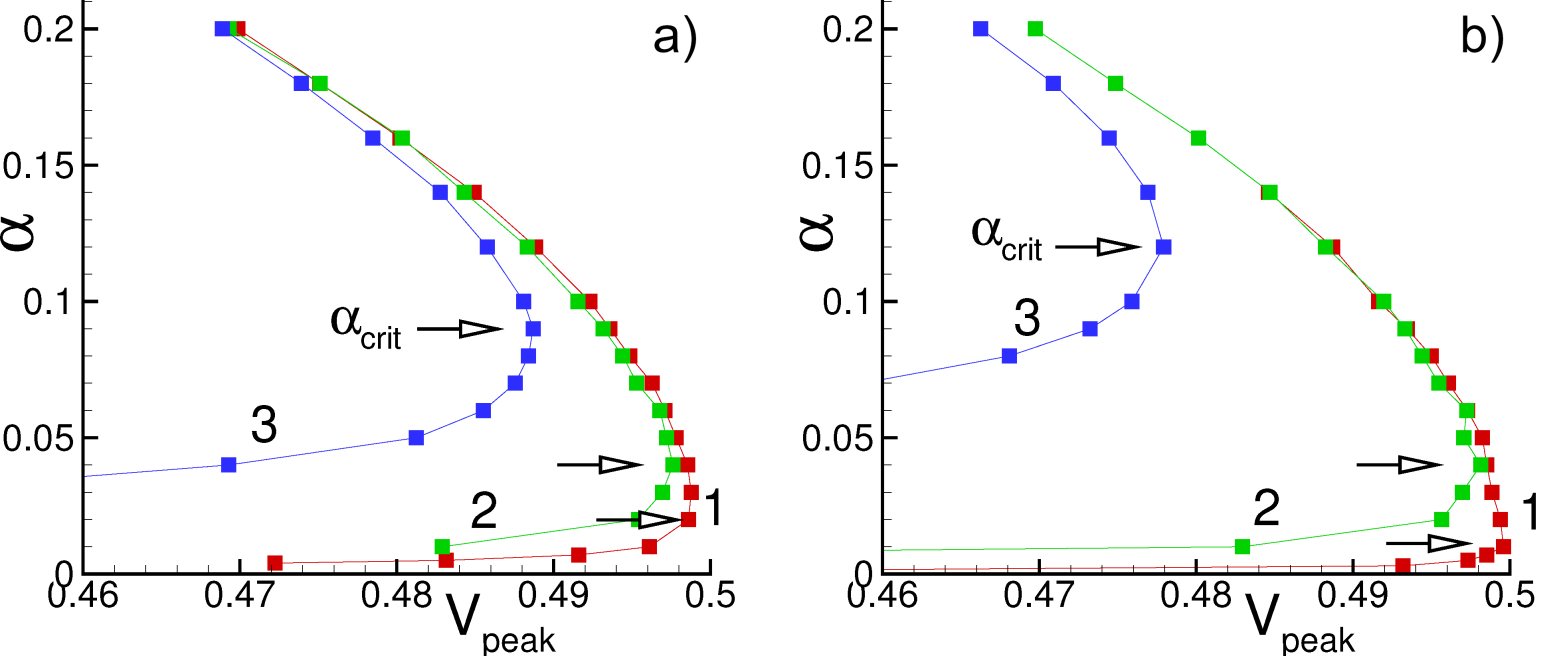
(a) Demonstration of ADD at different values of SOC parameter *r* at *G* = 0.05. Numbers indicate: 1 – *r* = 0.05; 2 – *r* = 0.1; 3 – *r* = 0.5; Arrows show critical α value, corresponded to the reversal in the α dependence. (b) Demonstration of ADD at different values of the Josephson-to-magnetic energy ratio *G* at *r* = 0.05. Numbers indicate: 1 – *G* = 0.01; 2 – *G* = 0.1; 3 – *G* = 1.

Figure 4a demonstrates results of numerical simulations of the 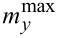 dependence on α at different values of the SOC parameter *r*.

It shows two specific features of ADD. First, with an increase in *r*, the critical value of *V*_peak_ decreases (the curve moves away from ω_F_). The second important feature is an increase of α_crit_, which is indicated by arrows in the figure.

Another model parameter that affects the phenomenon discussed in the present paper is the ratio *G* between Josephson energy and magnetic energy. Figure 4b demonstrates the results of numerical simulations of the 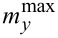 dependence on α at different values of *G*.

Similar to the effect of *r*, increasing *G* also causes the value of α_crit_ to increase. By changing the volume of the ferromagnetic layer, the ferromagnetic energy and, consequently, the value of *G* can be changed [6]. For small values of *G*, that is, a situation where the magnetic energy is much larger than the Josephson energy, the magnetic layer receives less energy, and its amplitude decreases in the *y* direction. Also, the maximum value of the oscillation frequency is closer to the magnetic frequency ω_F_.

### B. Dynamics and *I*–*V* characteristics of the φ_0_ junction at small values of system parameters

As it was discussed in [6,29–30], in the case of *G*, *r* and α ≪ 1, and *m_z_* ≈ 1, first three equations of the system in Equation 6 can be simplified. Taking into account φ = ω_J_*t* and neglecting quadratic terms of *m_x_* and *m_y_*, we get


[7]

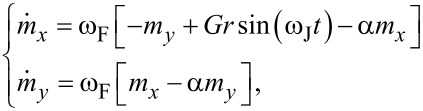



This system of equations can be written as the second-order differential equation with respect to *m_y_*,


[8]

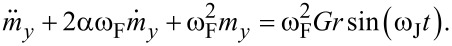



The corresponding solution for *m_y_* has the form


[9]

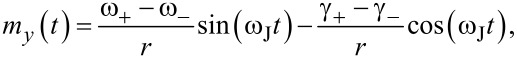



where


[10]

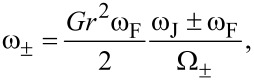



and


[11]

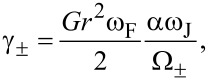



with Ω_±_ = (ω_J_ ± ω_F_)^2^ + (αω*_J_*)^2^ (see [6] and the corresponding erratum [31]).

When the Josephson frequency ω_J_ is approaching the ferromagnetic frequency ω_F_, *m_y_* exhibits damped ferromagnetic resonance. The differential resistance in the resonance region decreases, which is manifested in the *I*–*V* characteristics as a resonance branch [7].

Taking into account *rm_y_* ≪ 1, we rewrite the expression for the superconducting current as


[12]

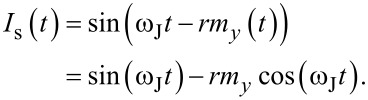



Using Equation 9 we obtain


[13]

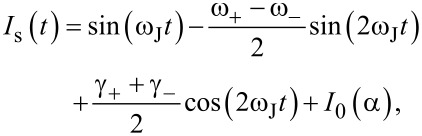



where


[14]

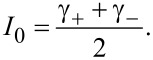



This superconducting current explains the appearance of the resonance branch in the *I*–*V* characteristics. The generated current *I*_0_ can be expressed through the amplitude of *m_y_* and the SOI parameter *r*,


[15]

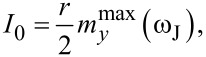



with 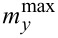
(ω_J_) being the frequency response of *m_y_*.

**Figure 5:**
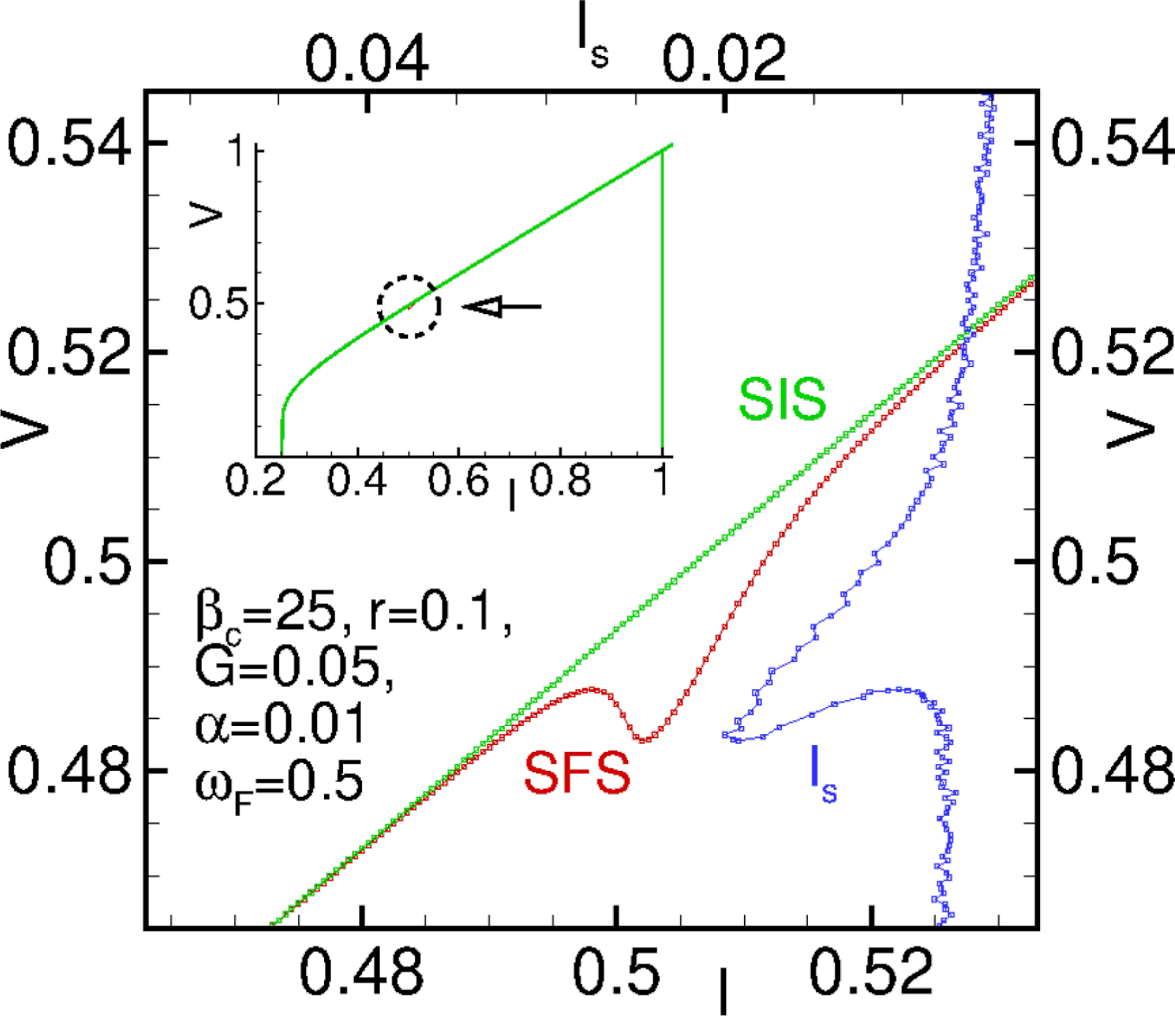
*I*–*V* characteristics of φ_0_ and SIS junctions and calculated average superconducting current through the φ_0_ junction.

At small model parameters α ≪ *Gr* ≪ 1 of a superconductor-ferromagnet-superconductor (SFS) φ_0_ Josephson junction, states with a negative differential resistance appear in the *I*–*V* characteristics in the FMR region. Due to the nonlinearity, the resonance peak is asymmetric. An increase of the nonlinearity leads to bistability (fold-over effect). The question appears if the states with a negative differential resistance are the origin of the fold-over and ADD. In order to clarify this question, we show in Figure 5 a part of the *I*–*V* characteristics of the φ_0_ junction together with the *I*–*V* characteristics of a superconductor-insulator-superconductor (SIS) junction in the ferromagnetic resonance region and the numerically calculated superconducting current through the φ_0_ junction. The total *I*–*V* characteristics are demonstrated in the inset to this figure.

We see the correlation of the fold-over effect in the superconducting current (blue) with the NDR part of the *I*–*V* curve. The peak in the superconducting current and the minimum of the *I*–*V* curve are at the same voltage value. So, both effects reflect the nonlinear features of the ferromagnetic resonance in the φ_0_ junction. However, in contrast to the fold-over and ADD effects, which begin to appear at relatively small deviations from the linear case, the nonlinearity in case of the NDR plays a more essential role.

We note that, in the resonance region for the considered limit of model parameters, the *m_y_* amplitude is coupled to the value of the superconducting current (see Equation 15). We stress the importance of the performed analysis demonstrating the analytical coupling of time-independent superconducting current and magnetization, reflecting the Duffing oscillator features of the φ_0_ junction.

As it is well known, the states with negative differential resistance appear in the *I*–*V* characteristics of Josephson structures in different physical situations. In particular, nonlinear superconducting structures being driven far from equilibrium exhibit NDR states [32]. The NDR states plays an essential role in applications related to terahertz radiation emission [33]. A detailed explanation of the different types of negative differential resistance in Josephson junctions (i.e., N-shaped and S-shaped) is introduced in [34]. The authors emphasize that the nonlinear behavior of the Josephson junction plays a key role in the NDR feature. In our case, the NDR states appear as a result of the nonlinearity of the system at small values of φ_0_ junction parameters, such as SOC, ratio between Josephson energy and magnetic energy, and Gilbert damping. We demonstrate these effects here by presenting results of detailed investigations of the NDR state at different system parameters and discuss the possibility of their control near the ferromagnetic resonance.

**Figure 6:**
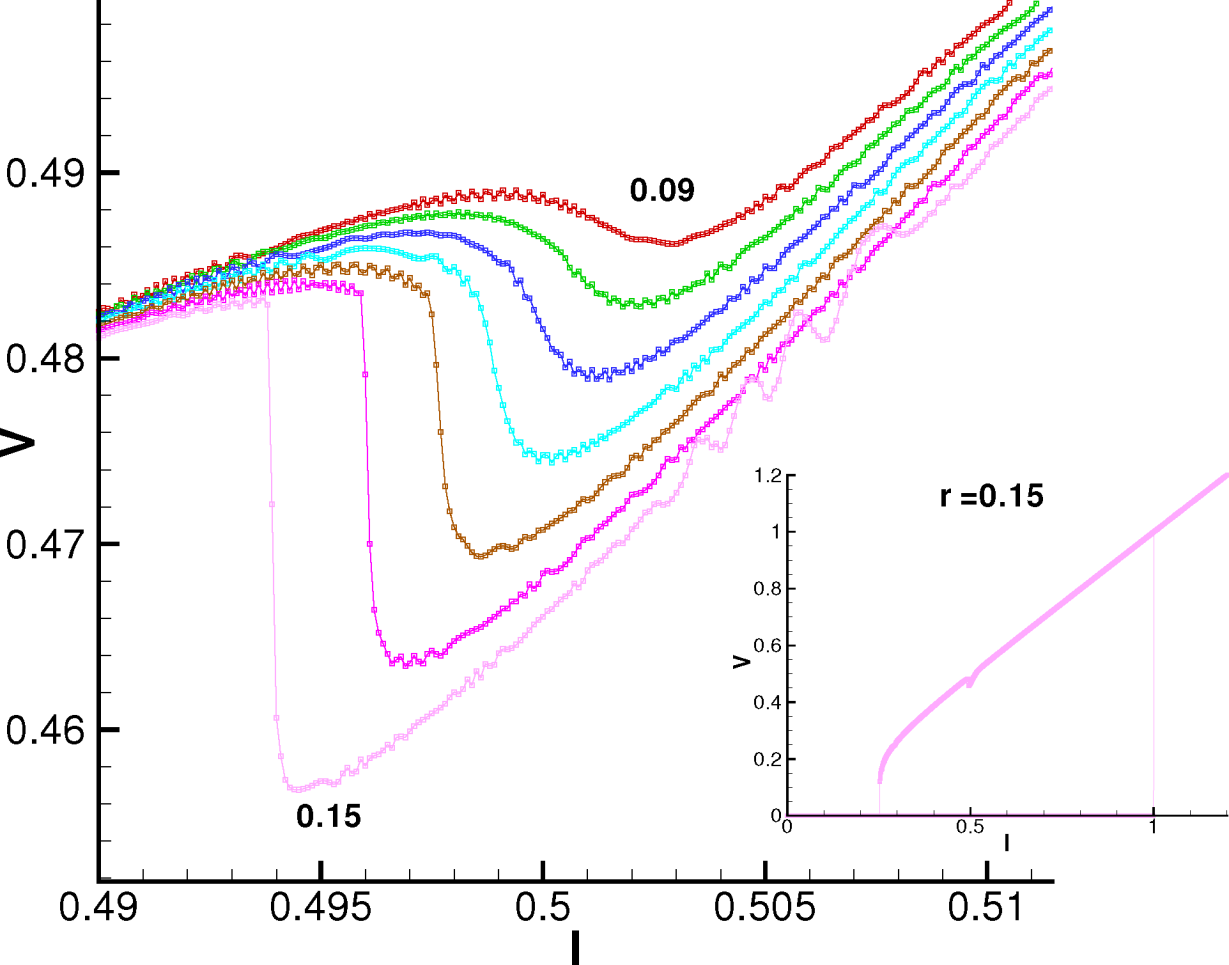
Enlarged parts of the *I*–*V* curves, in the resonance region at different values of the SOC parameter *r*, at α = 0.01 and *G* = 0.05. The numbers indicate the increase of *r* from 0.09 to 0.15 with an increment of 0.01. The inset shows the total *I*–*V* characteristics ar *r* = 0.15.

Figure 6 shows the effect of the spin–orbit coupling on the *I*–*V* characteristics at *G* = 0.05 and α = 0.01. We see the NDR feature, which is getting more pronounced with an increase in *r*. A further increase in *r* leads to a jump down in voltage and then practically linear growth of the *I*–*V* characteristics.

**Figure 7:**
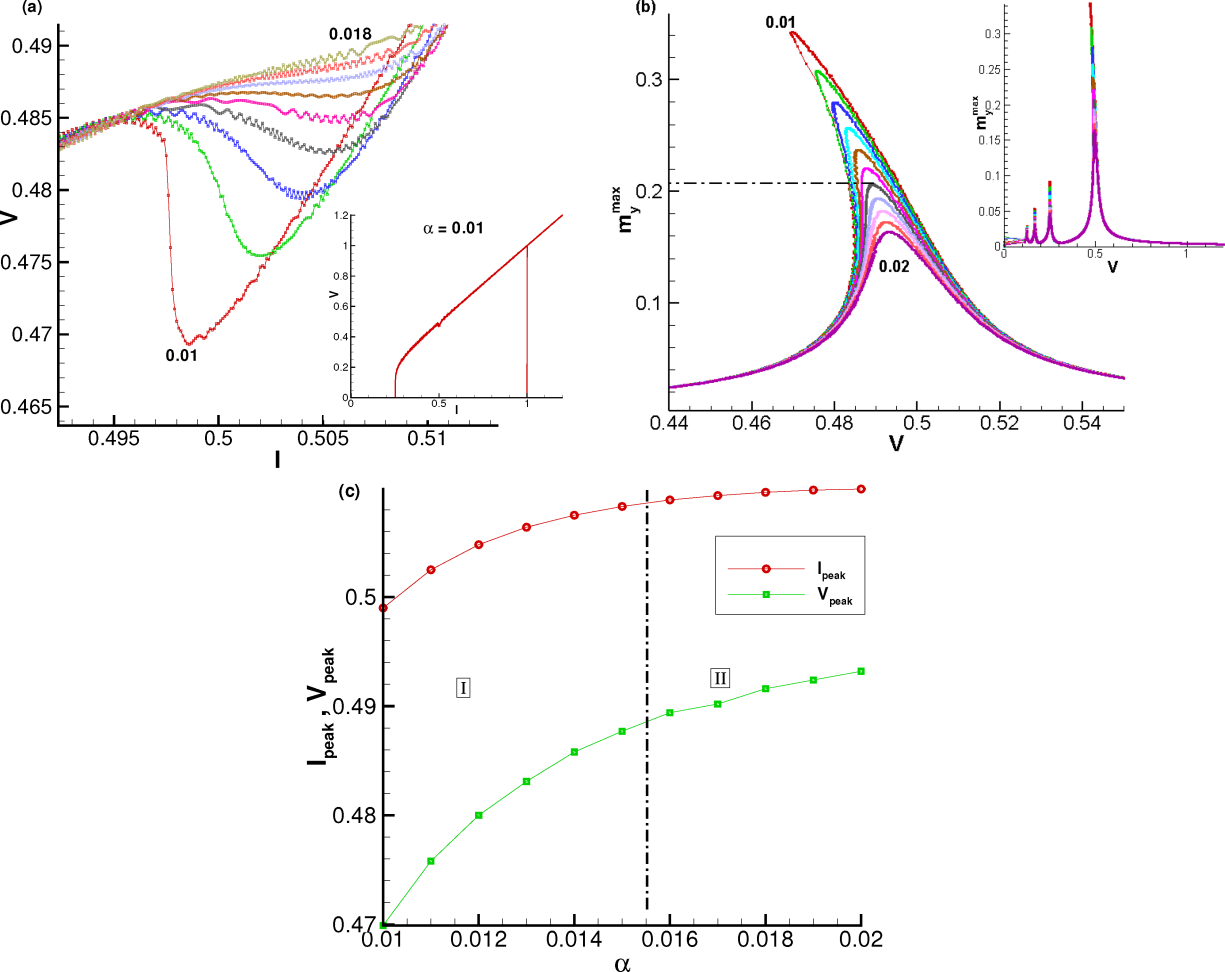
(a) Enlarged parts of the *I*–*V* curves at different values of α. (b) Voltage dependence of 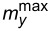 at different α. (c) α-Dependence of the resonance curve maximum in current (*I*_peak_) and voltage (*V*_peak_). The numbers indicate the value of α from 0.01 to 0.018 in (a) and from 0.01 to 0.02 in (b) by an increment 0.001. Results were obtained at *r* = 0.13 and *G* = 0.05.

An interesting question concerns the effect of Gilbert damping. Results of *I*–*V* characteristics simulations in the resonance region in a certain range of the damping parameter α at *G* = 0.05 and *r* = 0.13 are shown in Figure 7a. In this case, the most pronounced characteristic appears at α = 0.01. At *G* = 0.05 and *r* = 0.13, the range of α with pronounced NDR features is 0.01 ≤ α *<* 0.014.

The maximal amplitude 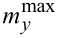 as a function of the voltage is shown in Figure 7b. Based on the results presented in Figure 7a and Figure 7b, we came to the important conclusion that the fold-over effect (bistability) and the NDR state have strong correlations and have the same origin related to the nonlinearity at small system parameters.

However, the anomalous damping dependence does not show a one-to-one correlation with either negative differential resistance or fold-over effect. The resonance peak positions of 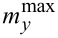 in bias current *I*_peak_ and in voltage *V*_peak_ as functions of α are demonstrated in Figure 7c. According to our results, we can divide the α interval into two regions (see Figure 7c). Region I includes the values of α where the NDR feature is present, while in region II it disappears. In region II the fold-over effect (bistability) disappears as well, but ADD is realized.

### C. Duffing oscillator features of the φ_0_ junction and critical damping

The system in Equation 6 is nonlinear and very complex. Hence, in order to provide an analytical study of dynamics of the φ_0_ junction, we need to derive an approximated equation for some limited values of model parameters. In [14], it was shown that the resonance curves demonstrate features of a Duffing oscillator, reflecting the nonlinear nature of the LLGJ system of equations. In this section, we present an analytical approach to describe the nonlinear dynamics of the φ_0_ junction and compare analytical results obtained from am approximated Duffing equation with numerical simulations of the total system in Equation 6. We show that in the limit of α ≪ *G* and *r* ≪ 1, we arrive at the Duffing oscillator. We start with the first three equations of Equation 6 for the magnetization components:


[16]

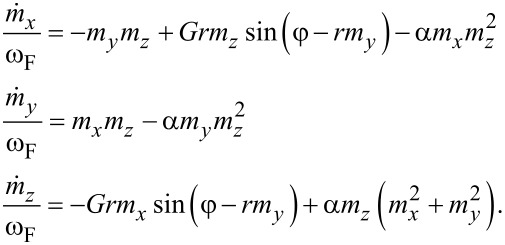



Simplifying this system of equations by the same procedure as it was done in [14], we can write equation for *m_y_* as


[17]

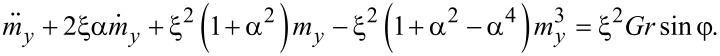



Finally, by neglecting the α^2^ and α^4^ terms, which are much smaller than 1, we come to the well-known Duffing equation,


[18]

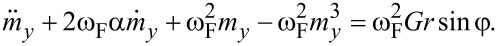



In the range of small parameter values, this Duffing equation can describe the dynamics of *m_y_*. We will have the full dynamics once we consider the coupling with the Josephson equation,


[19]

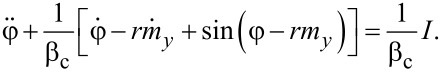



The system of Equation 18 and Equation 19 can replace the LLGJ equations in the limit of *G*, *r* ≪ 1 and *G*, *r* ≪ α.

Taking into account φ = ω_J_*t* we can write the analytically obtained frequency response for Equation 18,


[20]

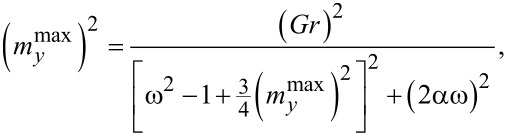



where ω = ω_J_/ω_F_. From Equation 20 we get


[21]

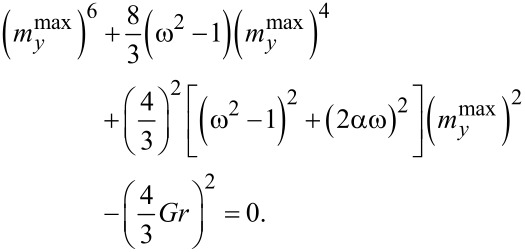



**Figure 8:**
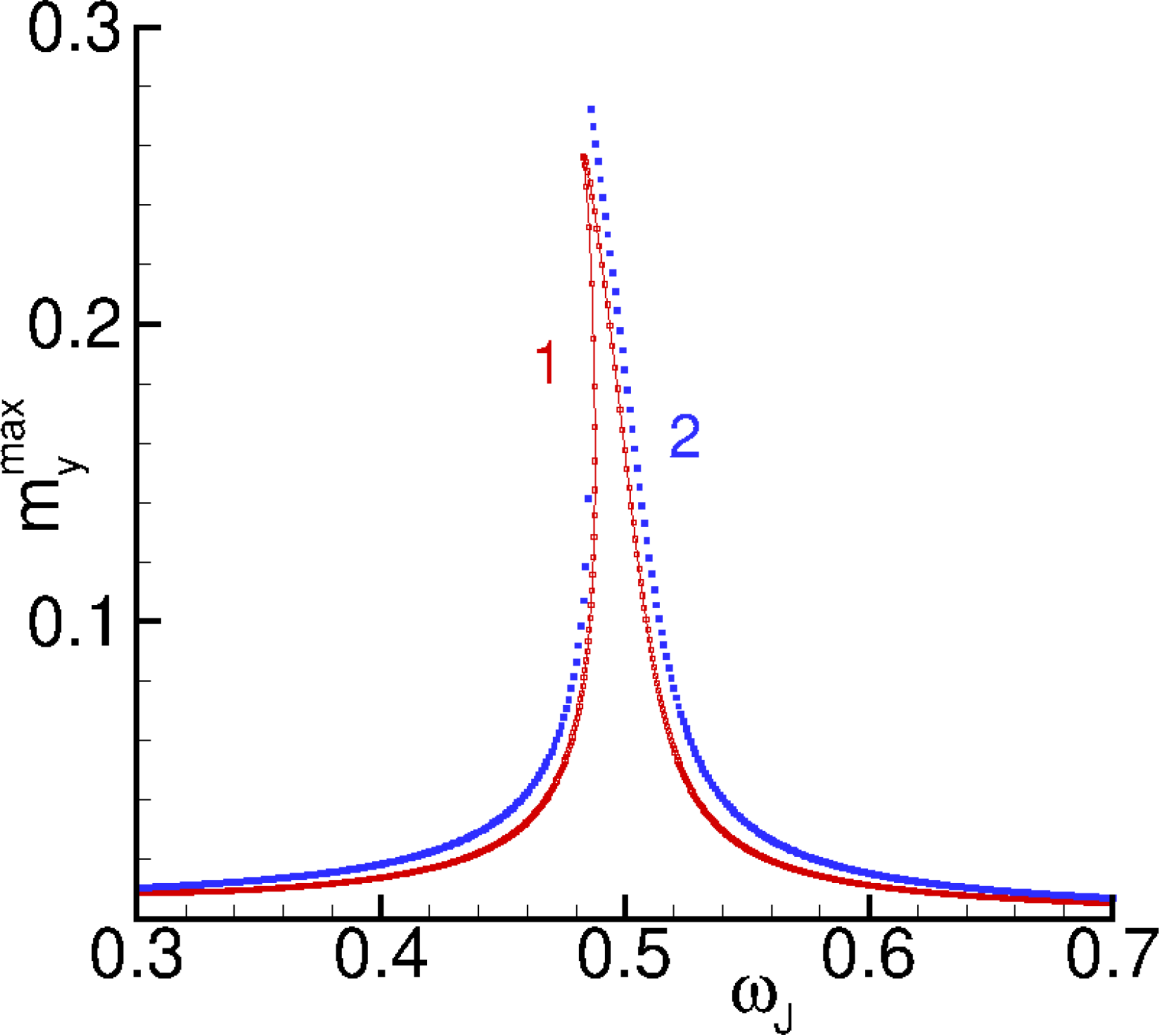
Numerically (red curve) and analytically (blue curve) calculated amplitude dependence of *m_y_*.

This equation allows one to determine analytically the frequency dependence of the 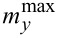 amplitude. To find it, we solve Equation 21 by the Newton method. Results of the analytical calculations (blue dots), corresponding to Equation 21, and the numerical solution (red dots), corresponding to the full system in Equation 6, are given in Figure 8.

We can see that they are close to each other, which proves the correctness of the chosen approximation. Both curves demonstrate an asymmetric resonance peak, which is common for a Duffing oscillator. When the role of the cubic term is getting larger, we observe a bistability of the resonance curve, which is usually called a fold-over effect. Note that the fold-over effect can be also achieved by decreasing the damping. This means that, by decreasing the dissipative term in Equation 18, we can increase the influence of the cubic term in this equation.

**Figure 9:**
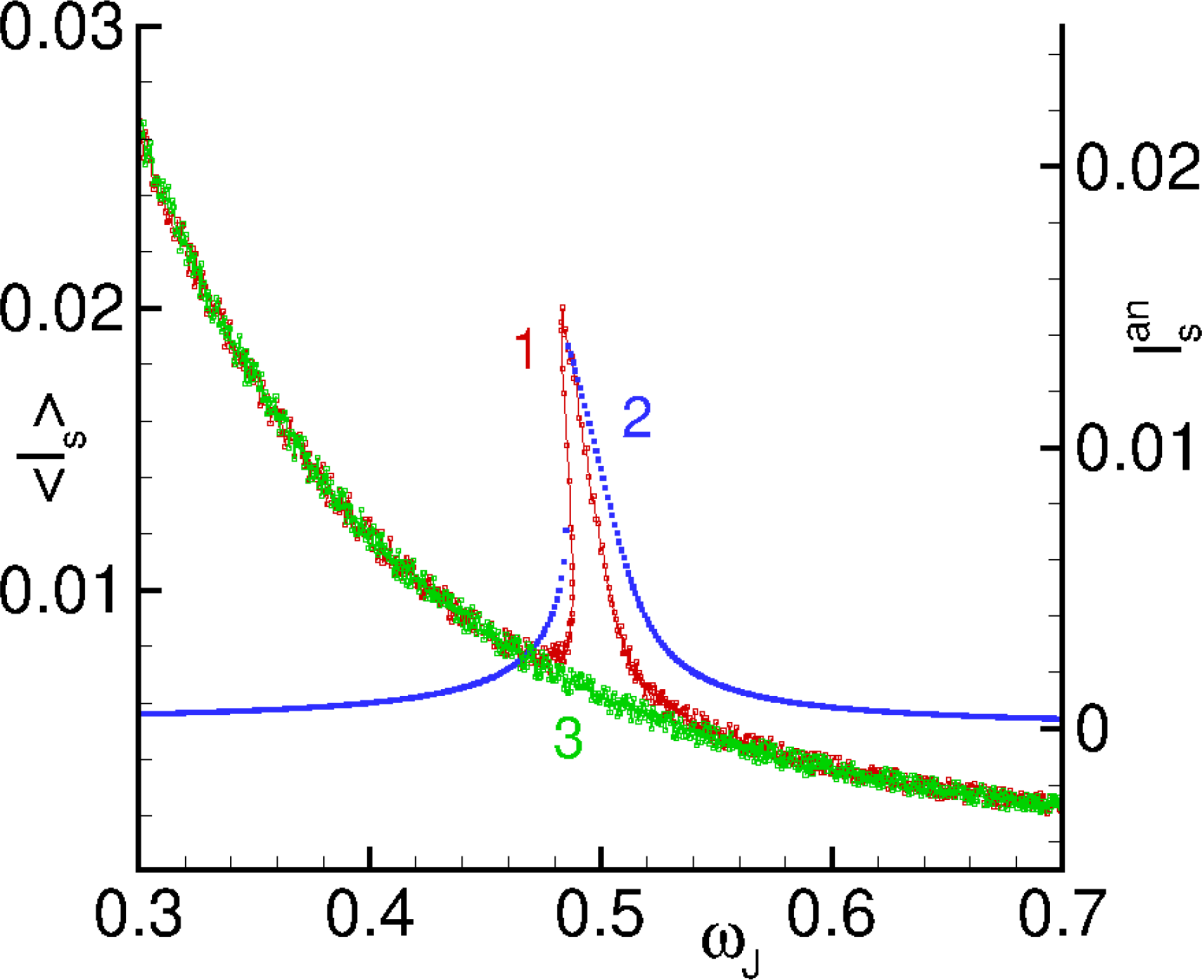
Numerically calculated superconducting current for SFS junction (plot 1) and analytical *I*_0_ (plot 2) and superconducting current for SIS junction (plot 3).

The comparison of analytically and numerically calculated superconducting currents as a function of the Josephson frequency is demonstrated in Figure 9. We note that in our normalization *V* = ω_J_.

We can see the manifestation of the asymmetric resonance peak in the frequency dependence of the superconducting current. So, the approximated system in Equation 7 reflects one of the main features of a Duffing oscillator.

**Figure 10:**
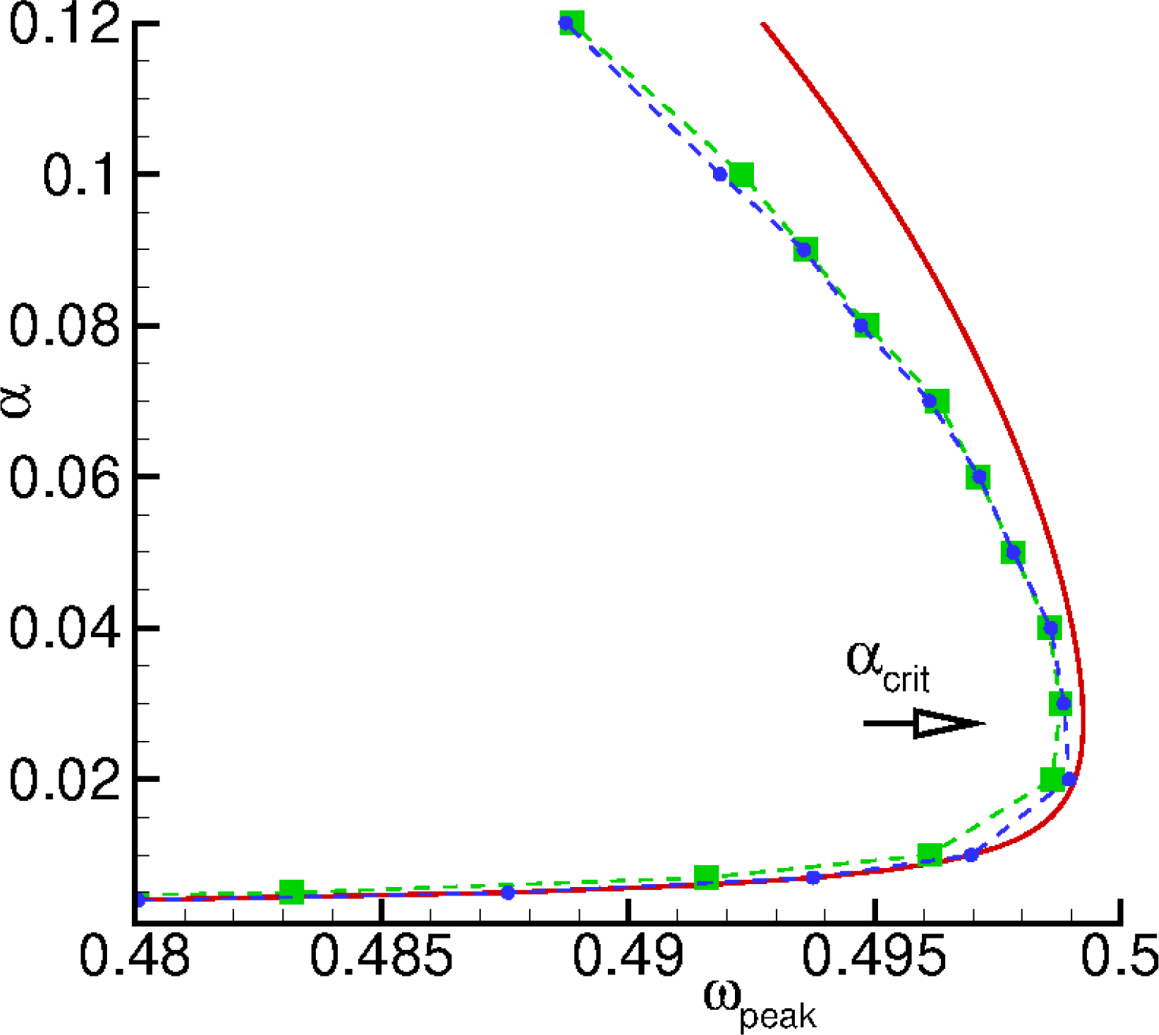
The dependence of the resonance maximum of 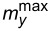
(*V*) on α in the damping parameter interval [0.001–0.12]. Green squares show results calculated numerically according to the full system in Equation 6, blue circles show results calculated numerically according to the generalized Duffing and Josephson equations (Equation 17 and Equation 19). The dashed line connects the symbols to guide the eyes. The solid line show the analytical dependence on α calculated according to Equation 22. All calculations have been carried out with β_c_ = 25, *G* = 0.05, *r* = 0.05, and ω_F_ = 0.5.

Figure 10 compares the anomalous damping dependence of the resonance peak of 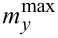
(*V*) calculated numerically according to the full LLGJ system in Equation 6 with the one calculated numerically according to the generalized Duffing model (Equation 17 and Equation 19). We see that in the damping parameter interval [0.001–0.2] the agreement of the dependences is sufficiently good.

Using Equation 18 with φ = ω_J_*t*, we can find (see Supporting Information File 1) a relation between the position of the resonance peak in the 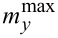
(*V*) dependence and the damping,


[22]

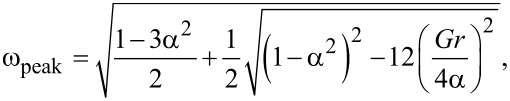



where 
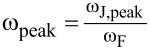
 determines the position of the resonance peak.

Equation 22 allows one to find the formula for the critical damping α_crit_, which is an important parameter determining the reversal point in damping dependence of the resonance peak in 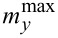
(*V*).

Taking into account Equation 22 we can write the equation regarding *Gr*/(4α) (see Supporting Information File 1),


[23]

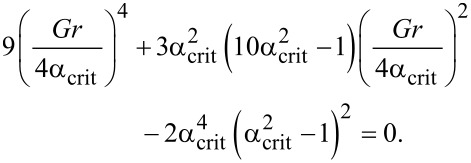



Using the approximation 10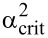 ≪ 1 and 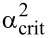 ≪ 1, it gives (see Supporting Information File 1)


[24]

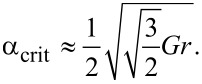



**Figure 11:**
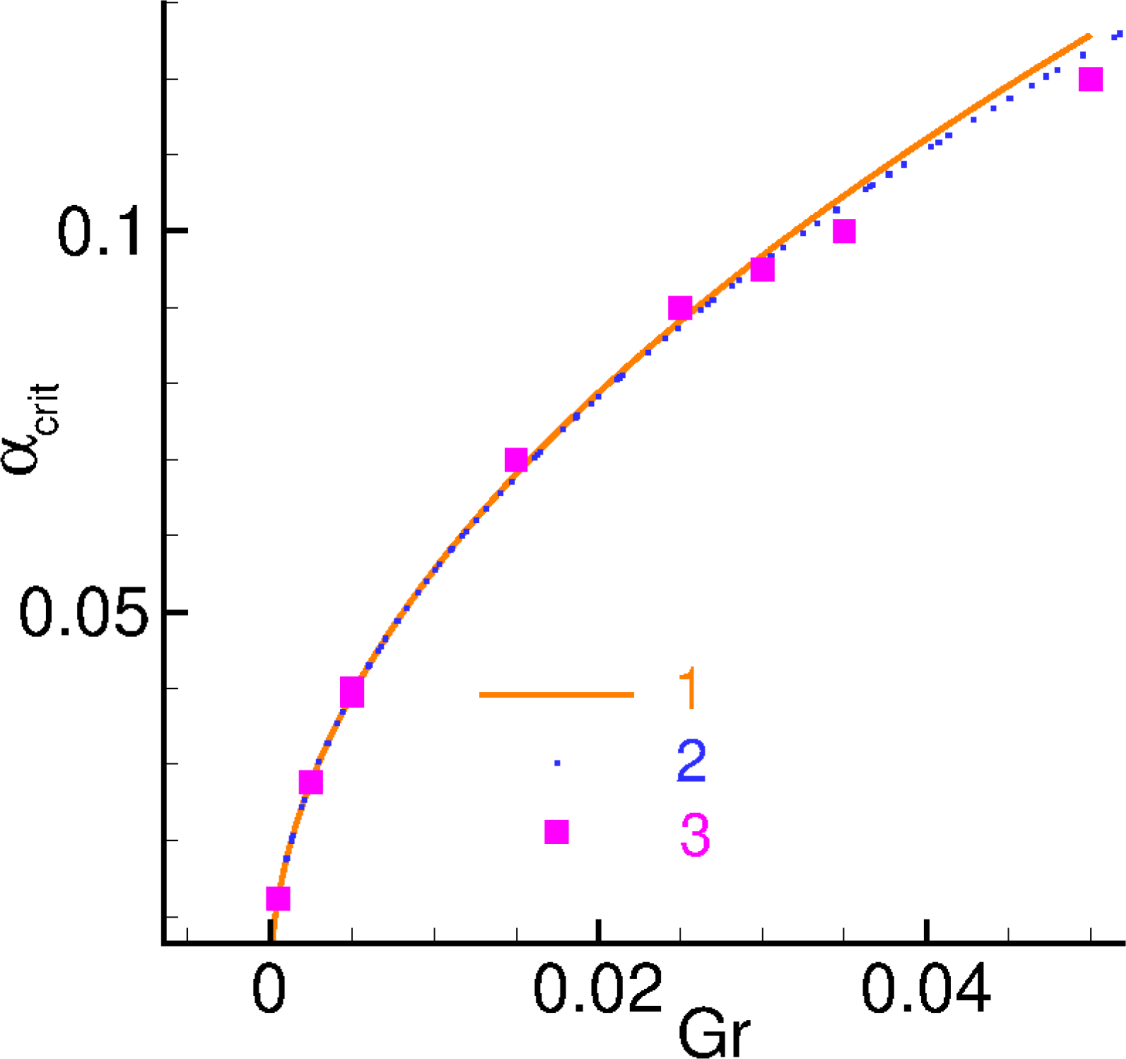
Numerical calculations according to Equation 6 (squares), analytical calculations according to Equation 23 (solid line), and approximated analytical calculations according to Equation 24 (dashed line).

Figure 11 presents a comparison of numerical and analytical results for α_crit_ as a function of *Gr* (Table 1).

**Table 1:** A comparison between the numerical and analytical values of α_crit_ at different values of *G* and *r*.

*G*	*r*	*Gr*	α_crit_, numerical	α_crit_, analytical

0.01	0.05	0.0005	0.0100	0.0123
0.05	0.05	0.0025	0.0300	0.0276
0.05	0.10	0.0050	0.0400	0.0391
0.05	0.30	0.0150	0.0700	0.0677
0.05	0.50	0.0250	0.0900	0.0874
0.10	0.05	0.0050	0.0391	0.0391
0.60	0.05	0.0300	0.0950	0.0958
0.70	0.05	0.0350	0.1000	0.1035
1.00	0.05	0.0500	0.1200	0.1237

There is a good agreement between numerical and analytical results of the calculations for small products of Josephson-to-magnetic energy ratio and spin–orbit interaction.

## Conclusion

The understanding of the nonlinear features of magnetization dynamics in superconductor–ferromagnet–superconductor Josephson junctions and their manifestation in the *I*–*V* characteristics has implications for superconductor spintronics and modern information technology. In φ_0_ junctions, the nonlinear features can affect the control of magnetization precession by the superconducting current and external electromagnetic radiation [28].

Here, using numerical and analytic approaches, we have demonstrated that at small values of the system parameters damping, spin–orbit interaction, and Josephson-to-magnetic energy ratio in φ_0_ junctions, magnetic dynamics is reduced to the dynamics of the scalar Duffing oscillator driven by the Josephson oscillations. We have clarified the role of the increasing superconducting current in the resonance region leading to the fold-over effect in the ferromagnet magnetization. We have demonstrated the parameter dependence of the anomalous ferromagnetic resonant shifting and the anomalous damping dependence due to the nonlinearity of the full LLGJ system of equations and its different approximations. We have derived an analytical expression for critical damping value. Also, we demonstrated the appearance of negative differential resistance in the *I*–*V* characteristics and the correlation with the occurrence of the fold-over effect in the magnetization of ferromagnet.

We have stressed that the manifestation of negative differential resistance is related to the nonlinear features of the system [34–35]. It was demonstrated that in the case of small model parameter values, the equation for the magnetic subsystem takes the form of the Duffing equation where the nonlinearity manifest itself as the cubic term. We have shown that the appearance of negative differential resistance in the *I*–*V* curve is related to the appearance of the fold-over effect in the 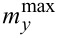
–*V* curve.

We believe that experimentally measured *I*–*V* characteristics of φ_0_ junctions with the manifestations discussed in detail here, would allow for close investigations of its nonlinear features important for superconductor electronics and spintronics.

## Supporting Information

File 1 Details of calculations for Equation 22 and Equation 24.
