# Supplementary material for: Nonlinear features of the superconductor–ferromagnet–superconductor φ0 Josephson junction in the ferromagnetic resonance region
Source: Beilstein J Nanotechnol. 2022 Oct 21;13:1155–66. doi: 10.3762/bjnano.13.97 (PMC9592964; doi:10.3762/bjnano.13.97)
Supplement: File 1 — Details of calculations for Equation 22 and Equation 24. [file Beilstein_J_Nanotechnol-13-1155-s001.pdf]

BEILSTEIN JOURNAL OF NANOTECHNOLOGY

Supporting Information

for

Nonlinear features of the superconductor–ferromagnet–superconductor φ_0 Josephson junction in the ferromagnetic resonance region

Aliasghar Janalizadeh, Ilhom R. Rahmonov, Sara A. Abdelmoneim, Yury M. Shukrinov and Mohammad R. Kolahchi

Beilstein J. Nanotechnol. **2022,** *13,* 1155–1166. doi:10.3762/bjnano.13.97

Details of calculations for Equation 22 and Equation 24

License and Terms: This is a supporting information file under the terms of the Creative Commons Attribution License (https://creativecommons.org/licenses/by/4.0). Please note that the reuse, redistribution and reproduction in particular requires that the author(s) and source are credited and that individual graphics may be subject to special legal provisions.
The license is subject to the *Beilstein Journal of Nanotechnology* terms and conditions: (https://www.beilstein-journals.org/bjnano/terms)

According to our approximation, at small values of the system parameters, the y component of magnetization can be determined by the general Duffing equation (Equation S1):

$$\frac{d^2 m_y}{dt^2} + 2\alpha\omega_F \frac{dm_y}{dt} + \omega_F^2 m_y - \omega_F^2 m_y^3 = \omega_F^2 G r \sin(\omega_J t). \tag{S1}$$

In Equation S1, α is a phenomenological damping constant, ω_F is the frequency of the ferromagnetic resonance, ω_J is the Josephson frequency, G is the ratio between Josephson energy and magnetic energy, and the parameter r determines the strength of the spin–orbit interaction.

In order to find frequency response function for Equation S1, we have performed the following procedure: We assume that the approximation solution of Equation S1 has the form

$$m_y = a \sin \omega_J t + b \cos \omega_J t, \tag{S2}$$

where a and b are functions of ω_J.

The first- and second-order derivatives of m_y are determined by

$$\begin{aligned} \frac{dm_y}{dt} &= a\omega_J \cos \omega_J t - b\omega_J \sin \omega_J t \\ \frac{d^2 m_y}{dt^2} &= -a\omega_J^2 \sin \omega_J t - b\omega_J^2 \cos \omega_J t. \end{aligned} \tag{S3}$$

Using trigonometric identities, the cube of m_y can be written as

$$m_y^3 = \frac{3}{4}(a^2 + b^2)[a \sin \omega_J t + b \cos \omega_J t]. \tag{S4}$$

Substituting Equation S3 and Equation S4 into Equation S1 and equating the coefficients at $\sin \omega_J t$ and $\cos \omega_J t$, we find

$$[\omega_F^2 - \omega_J^2 - \frac{3}{4}\omega_F^2 (m_y^{max})^2] a - 2\alpha\omega_F\omega_J b = \omega_F^2 G r$$
$$, 2\alpha\omega_F\omega_J a + [\omega_F^2 - \omega_J^2 - \frac{3}{4}\omega_F^2 (m_y^{max})^2] b = 0, \tag{S5}$$

where $(m_y^{max})^2 = a^2 + b^2$. Squaring both side of the system in Equation S5 and summing them, we get the frequency response function,

$$(m_y^{max})^2 = \frac{\left(\omega_F^2 G r\right)^2}{\left[\omega_J^2 - \omega_F^2 + \frac{3}{4}\omega_F^2 (m_y^{max})^2\right]^2 + \left(2\alpha\omega_F\omega_J\right)^2}. \tag{S6}$$

Introducing $\omega = \omega_J / \omega_F$, we can rewrite it in a simple form:

$$(m_y^{max})^2 = \frac{\left(G r\right)^2}{\left[\omega^2 - 1 + \frac{3}{4}(m_y^{max})^2\right]^2 + \left(2\alpha\omega\right)^2}. \tag{S7}$$

In Figure S1, the frequency dependence of the amplitude corresponding to the expression in Equation S7 for values of $\alpha = 0.015, 0.02, 0.03, 0.04$ are demonstrated.

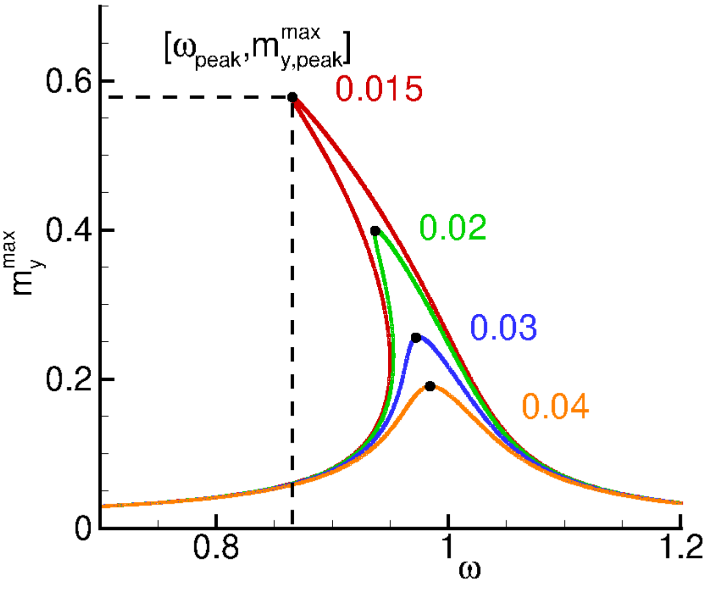

Figure S1: Frequency response in Equation S7, that is, m_y^{max} versus ω for $G = 0.05$, $r = 0.05$, and $\omega_F = 0.5$. Here, the numbers indicate the value of α.

To find the analytical anomalous damping dependence, we need to differentiate both side of Equation S7 with respect to ω and equate $d(m_y^{max})/d\omega$ to zero. We find

$$(m_{y,peak}^{max})^2 = \frac{4}{3}(1 - \omega_{peak}^2 - 2\alpha^2). \tag{S8}$$

Here $m_{y,peak}^{max}$ is the resonance peak and ω_{peak} is its position as shown in Figure S1. Finally, substituting Equation S8 into Equation S7, we obtain

$$\frac{4}{3}(1 - \omega_{peak}^2 - 2\alpha^2) = \frac{(Gr)^2}{(-2\alpha^2)^2 + (2\alpha\omega_{peak})^2}. \tag{S9}$$

After simplification of this expression, it can be written as

$$\frac{16}{3}\alpha^2(\alpha^2 + \omega_{peak}^2 - 3\alpha^2\omega_{peak}^2 - \omega_{peak}^4 - 2\alpha^4) = (Gr)^2. \tag{S10}$$

The solution of Equation S10 with respect to ω has the form

$$\omega_{peak} = \sqrt{\frac{1-3\alpha^2}{2} + \frac{1}{2}\sqrt{(1-\alpha^2)^2 - 12(\frac{Gr}{4\alpha})^2}}. \tag{S11}$$

Thus, it is an analytical expression for ADD and its plot is shown in Figure S2 for $G = 0.05$ and $r = 0.05$.

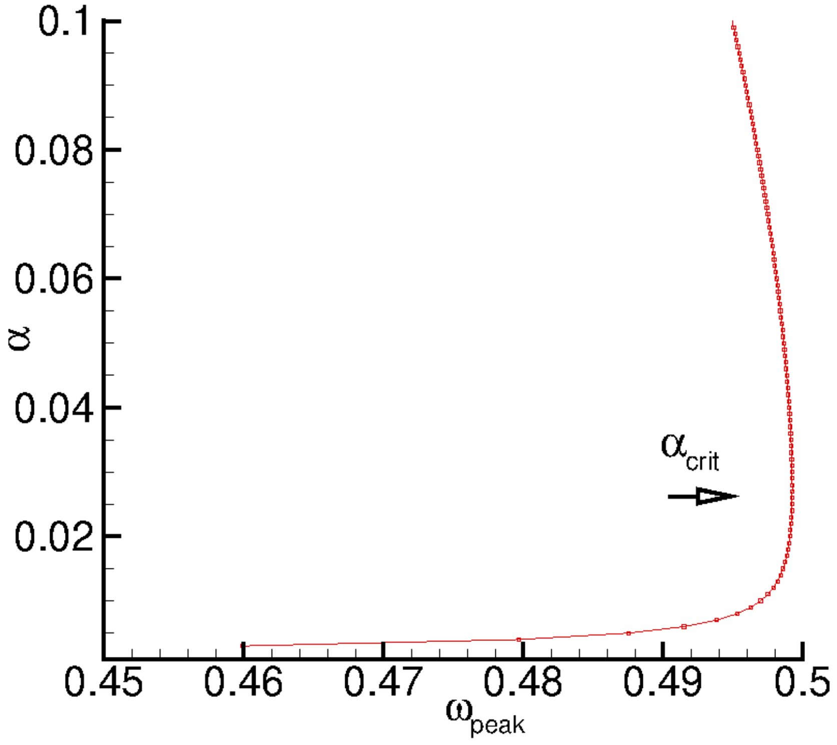

Figure S2: The resonance peak position depending on α for $G = 0.05$ and $r = 0.05$ and $\omega_F = 0.5$.

We can also find the expression for the critical value of α. To get it, we perform the following

procedure: After taking the derivative of Equation S10 with respect to α, we equate $\partial\omega_{peak}/\partial\alpha$ to zero and obtain the expression for α_{crit}:

$$6\alpha_{crit}^4 - 2\alpha_{crit}^2(1 - 3\omega_{peak}^2) - \omega_{peak}^2 + \omega_{peak}^4 = 0. \tag{S12}$$

Substituting ω_{peak} in Equation S12 from Equation S11, we get

$$9\left(\frac{Gr}{4\alpha_{crit}}\right)^4 + 3\alpha_{crit}^2(10\alpha_{crit}^2 - 1)\left(\frac{Gr}{4\alpha_{crit}}\right)^2 - 2\alpha_{crit}^4(\alpha_{crit}^2 - 1)^2 = 0. \tag{S13}$$

Taking into account $10\alpha_{crit}^2 << 1$ and $\alpha_{crit}^2 << 1$, Equation S13 can be rewritten as

$$9\left(\frac{Gr}{4\alpha_{crit}}\right)^4 - 3\alpha_{crit}^2\left(\frac{Gr}{4\alpha_{crit}}\right)^2 - 2\alpha_{crit}^4 = 0. \tag{S14}$$

The solution of Equation S14 has the form

$$\left(\frac{Gr}{4\alpha_{crit}}\right)^2 = \frac{3\alpha_{crit}^2 \pm \sqrt{9\alpha_{crit}^4 + 72\alpha_{crit}^4}}{18} \tag{S15}$$

or

$$\left(\frac{Gr}{4\alpha_{crit}}\right)^2 = \frac{\alpha_{crit}^2 \pm 3\alpha_{crit}^2}{6}. \tag{S16}$$

From here we can find

$$\alpha_{crit} = \frac{1}{2}\sqrt{\sqrt{\frac{3}{2}}Gr}. \quad \text{(S17)}$$
